# Beyond the Lungs: Extrapulmonary Effects of Non-Invasive and Invasive Ventilation Strategies

**DOI:** 10.3390/jcm14041242

**Published:** 2025-02-13

**Authors:** Pedro Leme Silva, Davide Chiumello, Tommaso Pozzi, Patricia Rieken Macedo Rocco

**Affiliations:** 1 Laboratory of Pulmonary Investigation, Carlos Chagas Filho Biophysics Institute, Federal University of Rio de Janeiro, Rio de Janeiro 21941, Brazil; pedroleme@biof.ufrj.br (P.L.S.); prmrocco@gmail.com (P.R.M.R.); 2 Department of Anesthesia and Intensive Care, ASST Santi Paolo e Carlo, San Paolo University Hospital Milan, 20142 Milan, Italy

**Keywords:** mechanical ventilation, cerebral perfusion, cardiac function, acute kidney injury, non-invasive respiratory support

## Abstract

**Background/Objectives**: Non-invasive respiratory support and invasive mechanical ventilation are critical interventions that can induce significant changes not only in the lungs but also in extra-pulmonary organs, which are often overlooked. Understanding the extra-pulmonary effects of non-invasive respiratory support and invasive mechanical ventilation is crucial since it can help prevent or mitigate complications and improve outcomes. This narrative review explores these consequences in detail and highlights areas that require further research. **Main Text**: Non-invasive respiratory support and invasive mechanical ventilation can significantly impact various extrapulmonary organs. For instance, some ventilation strategies can affect venous return from the brain, which may lead to neurological sequelae. In the heart, regardless of the chosen ventilation method, increased intrathoracic pressure (ITP) can also reduce venous return to the heart. This reduction in turn can decrease cardiac output, resulting in hypotension and diminished perfusion of vital organs. Conversely, in certain situations, both ventilation strategies may enhance cardiac function by decreasing the work of breathing and lowering oxygen consumption. In the kidneys, these ventilation methods can impair renal perfusion and function through various mechanisms, including hemodynamic changes and the release of stress hormones. Such alterations can lead to acute kidney injury or exacerbate pre-existing renal conditions. **Conclusions**: This review emphasizes the critical importance of understanding the extensive mechanisms by which non-invasive respiratory support and invasive mechanical ventilation affect extrapulmonary organs, including neurological, cardiovascular, and renal systems. Such knowledge is essential for optimizing patient care and improving outcomes in critical care settings.

## 1. Introduction

Non-invasive respiratory support [e.g., continuous positive airway pressure (CPAP), high-flow nasal oxygen (HFNO), and non-invasive ventilation (NIV)] and invasive mechanical ventilation are commonly applied in patients with acute respiratory failure to enhance gas exchange and reduce the work of breathing [1]. Both ventilatory strategies can also impact various organs besides the lungs, mainly the brain, heart, and kidneys [2]. Proper management of ventilation settings is crucial to mitigate potential organ complications. Understanding these mechanisms is essential for optimizing patient care and outcomes in critical care settings.

This narrative clinical review aims to provide a comprehensive understanding of how non-invasive respiratory support and invasive mechanical ventilation impact critical organ systems, emphasizing the need for careful management to minimize potential adverse effects. We will examine the interactions between these ventilation strategies and the central nervous, cardiovascular, and renal systems.

Studies for this narrative review were selected through a comprehensive literature search in major bibliographic databases (Cochrane Database of Systematic Reviews, PubMed, and Web of Science) to identify relevant research regardless of methodology. All selected full manuscript studies were in English, focusing on study design (e.g., pre-clinical studies, clinical trials, observational studies, and systematic reviews) and solely on the pulmonary and extrapulmonary effects of non-invasive and invasive ventilation strategies. Studies were then reviewed for relevance and quality, focusing on those that provided significant insights into the mechanisms and clinical outcomes of non-invasive respiratory support and invasive mechanical ventilation. Table 1 summarizes the possible physiological consequences of IMV according to each organ and the respective suggested practices.

## 2. Non-Invasive Respiratory Support

Non-invasive respiratory support is utilized for managing acute respiratory failure due to hypoxemic and/or hypercapnic conditions [18,19], as well as chronic hypercapnic respiratory failure [20]. This support is delivered through various devices, including bilevel positive airway pressure (BiPAP), CPAP, HFNO, and NIV. Each device employs different interfaces—such as nasal cannulas, oral masks, full-face masks, and helmets—each with its own advantages and disadvantages for patients.

For non-invasive respiratory support to be effective, several principles must be followed, irrespective of the device or interface used. The patient must retain an active respiratory drive, and the support should enhance respiratory comfort. Effectiveness is often gauged by a reduction in respiratory rate and a decrease or absence of accessory respiratory muscle use, among other respiratory parameters [21].

Non-invasive respiratory support can also have extrapulmonary effects on neurological, cardiovascular, and renal systems. These effects may be overlooked because patients receiving non-invasive respiratory support are often less monitored. Such unnoticed adverse effects could contribute to poorer outcomes in critically ill patients.

## 3. Neurological System

### 3.1. Non-Invasive Respiratory Support

The breathing pattern is primarily regulated by the brainstem and suprapontine structures. Brainstem phasic neurons control the ventilatory rhythm, adjusting ventilation to meet the body’s metabolic needs [22]. However, disruptions in this automatic control can occur due to interference from suprapontine structures or premotor cortical areas [23].

Raux et al. investigated the hypothesis that ventilator asynchrony during NIV would activate premotor cortical areas. They used inspiratory-related premotor potentials as the primary endpoint. Their study revealed that individual-ventilator asynchrony elicited premotor potentials, indicating activation of the premotor cerebral cortex. This suggests that the brain must exert additional effort to manage the mismatch, potentially increasing cognitive load and discomfort. The presence of premotor action potentials during discomfort suggests that suprapontine processes may influence ventilatory drive [22,24].

In patients with chronic obstructive pulmonary disease (COPD), the long-term effects of NIV were assessed to determine if it would alter the excitability of intracortical pathways [24]. NIV significantly reduced the work of breathing and was associated with a marked decrease in the normalized amplitude of the diaphragm motor-evoked potential in response to transcranial magnetic stimulation. However, NIV did not affect the excitability of intracortical inhibitory or facilitatory pathways, as measured by paired stimulation. The authors suggested that the absence of change in intracortical circuits might be attributed to altered plasticity capacity due to chronic exposure to blood gas imbalances or load-capacity imbalances in the respiratory muscles experienced by COPD patients.

Apart from gas imbalances and respiratory muscle disadvantages, delirium may occur during NIV. The onset of delirium in critically ill patients is well documented as a significant factor contributing to poor clinical outcomes [25]. A prospective, multicenter observational study reported a high incidence of delirium (36%) among patients with hypoxic acute respiratory failure in the ICU [7]. Key risk factors independently associated with delirium onset within the first week of ICU admission included advanced age, cancer, sepsis, and elevated SOFA, Borg, and PRE-DELIRIC scores at admission [7,8]. Whether hypoxic events during the ICU stay are directly associated with delirium remains to be elucidated in patients with multiple injuries [9]. In addition, the excessive use of sedative-hypnotic medication can lead to delirium in brain injury patients [26]. Moreover, the presence of delirium was found to adversely affect the success of NIV and overall clinical outcomes [7,27]. Despite conflicting evidence and studies in noncritically ill patients identifying significant adverse effects, antipsychotic agents remain the most common treatment for ICU delirium [10].

Patients with acute brain injury often require invasive mechanical ventilation for airway protection, impaired respiratory drives, and pulmonary edemas [28]. In extubated patients who required reintubation (up to 20%) [29], a recent study proposed the use of HFNO and NIV [30]. However, a prospective, multicenter cohort study found that HFNO and NIV did not significantly reduce the need for reintubation in brain-injured patients undergoing weaning from mechanical ventilation [31].

### 3.2. Invasive Mechanical Ventilation

The adequacy of respiratory rate and tidal volume (V_T_) during invasive mechanical ventilation is crucial for maintaining systemic CO_2_ levels within normal ranges. Variations in CO_2_ levels can significantly affect vascular reactivity throughout the body, particularly impacting the central nervous system. Cerebral blood flow (CBF) is influenced by the pressure differential between the arterial and venous sides of cerebral circulation and is inversely proportional to cerebral vascular resistance. Since direct measurement of venous pressure is challenging, intracranial pressure (ICP) is used as a surrogate to estimate cerebral perfusion pressure (CPP), calculated as the difference between mean arterial pressure and ICP.

CBF is closely linked to regional cerebral metabolism and is highly responsive to CO_2_ levels. Increased CO_2_ tension causes cerebral arteries to relax, with localized changes in CO_2_ or pH potentially altering vascular diameter [32]. Both endothelial/smooth muscle cells and extravascular cells, including perivascular nerve cells, neurons, and glia, contribute to these changes. For every mmHg change in arterial CO_2_ (PaCO_2_), CBF typically changes by approximately 3% within the range of 20 to 60 mmHg. Therefore, hypoventilation leading to hypercapnia results in vasodilation and increased CBF, while hyperventilation causes vasoconstriction and reduced CBF [33,34].

Hyperventilation has been used to manage severe traumatic brain injury (TBI) for over 40 years. A 0.5 mL change in blood volume can alter ICP by 1 mmHg. However, the vasoconstrictive effect of hyperventilation diminishes after 24 h as perivascular pH normalizes [35]. Despite its routine use in acute TBI, evidence from randomized clinical trials supporting hyperventilation is limited [36], with some studies suggesting better outcomes in patients who were not hyperventilated [37]. Comparative studies between hyperventilation and mannitol have found that moderate hyperventilation (reducing end-tidal CO_2_ by 5 mmHg) decreases CBF, while mannitol significantly, though moderately, improves cerebral perfusion [38].

In a secondary analysis of the ENIO study, Robba et al. [3] found that PaCO_2_ values were generally maintained within normal to mildly hypocapnic ranges during early ICU admission. Both profound hypocapnia (PaCO_2_ < 26 mmHg) and hypercapnia (PaCO_2_ > 45 mmHg) were associated with increased mortality. The impact of PaCO_2_ on in-hospital mortality varied with the type of acute brain injury, with mild hypocapnia (PaCO_2_ 32–35 mmHg) being better tolerated in TBI and intracranial hypertension compared to subarachnoid hemorrhage and ischemic stroke. Conversely, another study [4] indicated increased mortality in severe TBI patients with end-tidal CO_2_ (ETCO_2_) values below 35 mmHg, underscoring differences in patient populations and clinical practices.

The systemic inflammatory response is crucial in the development of pulmonary failure following acute brain injury [39]. Post-brain injury, intracranial inflammatory responses involve pro-inflammatory cytokines such as interleukin (IL)-1, IL-6, IL-8, and tumor necrosis factor (TNF), which increase lung susceptibility to further injury [40]. Protective ventilation strategies, including low V_T_, moderate-to-high positive end-expiratory pressure (PEEP), and recruitment maneuvers with permissive hypercapnia, may influence the progression of acute brain injury [41]. One multicenter prospective observational study found that neurologic patients were ventilated with similar V_T_s of approximately 9 ± 5 mL/kg but exhibited lower respiratory rates and PEEP levels compared to non-neurologic patients. The study also observed higher mortality rates among stroke patients, particularly those with hemorrhagic stroke, which may be attributed to underlying neurologic dysfunction [2].

High V_T_ (30 mL/kg) has been associated with increased cortical and thalamic c-fos expression compared to low V_T_ (8 mL/kg) at the same PEEP level [42]. Invasive mechanical ventilation has also been linked to selective hippocampal neuronal apoptosis, primarily through afferent vagus signaling [5]. Even short periods of high-V_T_ ventilation, followed by protective ventilation strategies, can result in detectable thalamic injury [43].

The optimal PEEP level for patients with brain injury remains unclear. A study comparing low (0 cmH_2_O) and moderate (8 cmH_2_O) PEEP levels found that moderate PEEP did not significantly alter most exhaled breath condensate (EBC) mediators but did reduce the systemic inflammatory response [6].

In a recent multicenter, open-label, controlled clinical trial, 190 adult patients with acute brain injury were randomized to receive either a lung-protective ventilatory strategy [V_T_ = 6.6 ± 0.8 mL/kg predicted body weight (PBW), PEEP = 8 ± 1 cmH_2_O] or a conventional strategy [V_T_ = 8.5 ± 1.1 mL/kg PBW, and PEEP = 5 ± 1 cmH_2_O] [44]. The primary outcome was a composite endpoint of death, ventilator dependency, and acute respiratory distress syndrome (ARDS) at 28 days. Using an intention-to-treat analysis, the composite outcome occurred in 61.5% of patients in the lung-protective group compared to 45.3% in the conventional group (*p* = 0.025). Mortality rates were 28.9% versus 15.1% (*p* = 0.02), ventilator dependency was 42.3% versus 27.9% (*p* = 0.039), and the incidence of ARDS was 30.8% versus 22.1% (*p* = 0.179), respectively. The study was terminated early due to funding constraints. There are some points that need to be discussed regarding the PROLABI trial. The trial stopped with 190 patients, 36% of the planned number according to the sample-size calculation (524 patients). Underpowered trials are classically subjected to type II error as acknowledged by the authors, but also may elicit type I error, where the detection of an effect might be due to chance. This may raise the possibility that enrolling more patients would achieve no difference between lung-protective ventilatory or conventional strategies. In addition, other issues have been discussed elsewhere [45], such as the robustness of results on ARDS development [46], once-daily recordings, very narrow differences between some ventilator variables, and lack of sedation and neuromuscular blocker protocol description.

## 4. Cardiovascular System

### 4.1. Non-Invasive Respiratory Support

The heart and lungs are anatomically close within the thorax, with the lungs serving as a conduit between the right and left heart chambers, establishing a significant interdependence between these organs. Both spontaneous breathing and invasive mechanical ventilation induce changes in intrapleural and intrathoracic pressure as well as lung volume, which can independently affect cardiovascular function by altering atrial filling (preload), ventricular emptying (afterload), heart rate, and myocardial contractility.

Extensive literature supports the use of non-invasive respiratory support for managing cardiogenic pulmonary edema [47,48]. The main mechanisms include the following:

1. Restoration of functional residual capacity (FRC): non-invasive respiratory support helps restore FRC, reducing shunt and improving oxygenation [49].
2. Increase in pleural pressure: elevated pleural pressure reduces left ventricular afterload (the difference between left ventricular systolic pressure and pleural pressure) without compromising the cardiac index [50].
3. Reduction in left ventricular end-diastolic volume (Preload): this effect is particularly beneficial in patients with preserved left ventricular function, as it decreases preload and consequently the left ventricular end-diastolic volume [51].

In summary, while non-invasive respiratory support can alter cardiovascular dynamics by increasing right atrial pressure and reducing venous return, it also provides therapeutic benefits in conditions such as cardiogenic pulmonary edema by improving oxygenation and reducing cardiac workload. These interactions highlight the importance of understanding the cardiovascular effects of non-invasive respiratory support in critically ill patients.

### 4.2. Invasive Respiratory Support

Spontaneous inspiration generates negative pleural pressure, reducing intrathoracic pressure. In contrast, invasive mechanical ventilation increases ITP and right atrial pressure due to positive pressure ventilation, regardless of PEEP. Each passive mechanical breath elevates ITP, which affects the end-diastolic volume and compliance of both the right and left ventricles (RV and LV, respectively) [11,12]. Increased ITP also reduces vena cava flow and right ventricular dimensions, leading to an elevated transseptal pressure gradient. This shift results in a rightward movement of the septum, increasing LV volume and potentially enhancing stroke volume [11].

The cardiovascular system is also significantly affected by changes in pulmonary vascular resistance (PVR) related to lung volume. PVR is lowest at FRC and increases at both higher and lower lung volumes [13]. Elevated PVR at extreme lung volumes may result from reduced distending pressure in small vessels not exposed to intrapleural pressure, decreased distensibility of pulmonary vessels, or changes in vascular geometry.

A study involving obese patients with ARDS [52] demonstrated that high PVR can be mitigated with appropriate lung recruitment, providing lung overdistension is avoided and hemodynamic stability is maintained. This underscores the importance of optimal lung recruitment for improving cardiovascular outcomes during positive pressure ventilation. A recent physiological study on ARDS patients [53] found that PEEP increased PVR only when it caused significant lung distension; however, PEEP had no effect when associated with lung recruitment [53].

## 5. Renal System

### 5.1. Non-Invasive Respiratory Support

Acute kidney injury in critically ill patients can arise from various mechanisms, including hemodynamic instability, which decreases renal perfusion and increases endothelial cell injury, thus leading to renal dysfunction, systemic inflammatory responses, and hypoxemia (given that renal cells are highly sensitive to oxygen levels). Despite these known risks, the specific role of non-invasive respiratory support in AKI remains unclear.

Evidence suggests that non-invasive respiratory support may be associated with a lower incidence of AKI compared to invasive mechanical ventilation. For instance, a clinical trial involving 64 patients with acute hypoxemic respiratory failure reported a 9% incidence of AKI in the NIV group versus 16% in the invasive mechanical ventilation group [54]. Additionally, a retrospective study found that the presence and severity of chronic kidney disease did not significantly impact the duration of NIV in patients with acute hypercapnic respiratory failure [55]. Conversely, preoperative AKI, classified as KDIGO Stage 2–3, was an independent predictor of prolonged NIV use following extubation in ICU patients after cardiac surgery [56]. Prolonged NIV was linked to poorer patient outcomes, including a higher risk of postoperative complications and extended ICU and hospital stays.

While retrospective studies offer some insights into the relationship between non-invasive respiratory support and renal outcomes, there remains a significant gap in understanding the full impact of non-invasive respiratory support on kidney function.

### 5.2. Invasive Respiratory Support

Patients with AKI are significantly more likely to develop respiratory failure requiring mechanical ventilation compared to those without AKI. The need for mechanical ventilation in AKI patients is an independent predictor of mortality, with studies showing mortality rates as high as 89% associated with this requirement [57].

Intra-abdominal pressure (IAP) is another critical factor influencing renal hemodynamics during positive pressure ventilation. Elevated IAP, which can result from positive pressure ventilation, respiratory system elastance, or pre-existing abdominal conditions such as high-volume fluid infusion, may impair microvascular blood flow and exacerbate kidney edema due to compromised venous drainage. Several well-established risk factors contribute to elevated IAP, including abdominal surgery, major trauma, gastroparesis, gastric distention, ileus, acute pancreatitis, damage control laparotomy, massive fluid resuscitation or positive fluid balance, and mechanical ventilation. One approach to mitigate these effects is the application of PEEP. However, studies have shown that PEEP levels up to 15 cmH_2_O do not prevent the decline in functional residual capacity induced by intra-abdominal hypertension (IAH) at 18 mmHg and are, in fact, associated with reduced oxygen delivery due to diminished cardiac output [14]. In a subsequent study utilizing a porcine model of IAH with healthy lungs, PEEP was titrated to match IAP. While end-expiratory lung volume was maintained, no improvement in arterial oxygenation was observed, and cardiac output decreased [58]. Conversely, in the setting of acute lung injury, PEEP titrated to match IAP reduced shunt and dead-space fractions and improved respiratory system elastance, primarily by decreasing chest wall elastance [15].

During invasive mechanical ventilation, cardiac output, urinary output, and sodium excretion may reduce depending on PEEP levels [59]; however, these effects can be reversed when PEEP is withdrawn.

Clinical evidence supports a link between mechanical ventilation and the development of AKI. A meta-analysis assessing the impact of V_T_ and PEEP on AKI in critically ill adults revealed increased odds of AKI with invasive mechanical ventilation. However, neither V_T_ nor PEEP were directly linked to AKI risk [60]. Notably, the ARDS Network trial found that lower V_T_ (6 mL/kg) was associated with more renal failure-free days compared to higher V_T_ (12 mL/kg) [16]. In contrast, the EXPRESS study found no significant difference in renal failure-free days between low- and high-PEEP groups [17].

In summary, invasive mechanical ventilation has complex effects on renal function, involving hemodynamic changes, inflammatory responses, and systemic factors. These interactions contribute to the risk of AKI in critically ill patients undergoing mechanical ventilation.

## 6. Conclusions

**Figure 1:**
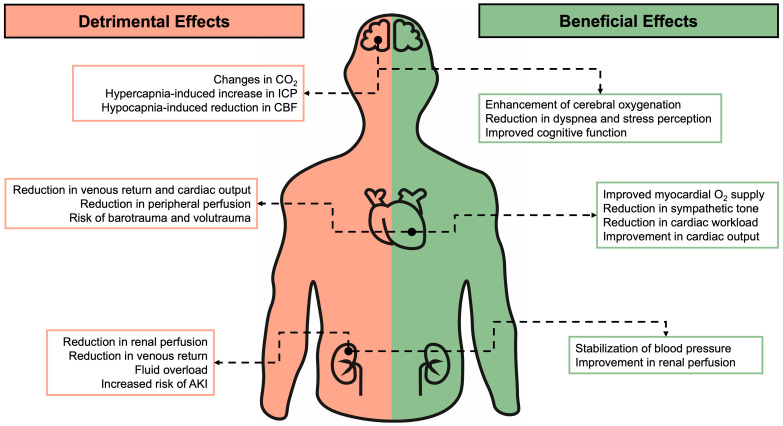
Beneficial (green) and detrimental (red) effects of non-invasive and invasive respiratory support according to organ involvement. CO_2_: carbon dioxide partial pressure. ICP: intracranial pressure; CBF: cerebral blood flow; O_2_: oxygen; AKI: acute kidney injury.

This review highlights the critical importance of understanding the comprehensive effects of both non-invasive respiratory support and invasive mechanical ventilation on extra-pulmonary organs (Figure 1). Clinicians can take several steps to prevent extrapulmonary complications during invasive and non-invasive ventilation by adopting a proactive and multidisciplinary approach, including the optimization of ventilatory settings, monitoring and management of cardiovascular effects, prevention of neurological complications, and protection of renal function. The use of personalized approaches based on individual patient characteristics, underlying conditions, and response to mechanical ventilation strategies should be adopted to avoid extrapulmonary complications and improve outcomes in patients requiring invasive and non-invasive ventilation. Working with a multidisciplinary team, including respiratory therapists, critical care nurses, and physical therapists, is important to ensure overall care.

## Figures and Tables

**Table 1:** Suggested practices for optimizing ventilation strategies in brain, heart, and kidney.

Organs	Possible Physiological Consequences of IMV	Suggested Practices
Brain	Changes in CO_2_ (1 mmHg change in PaCO_2_, CBF changes by 3%)	Mild hypocapnia and normocapnia (PaCO_2_ 32–35 mmHg) was well tolerated in TBI and intracranial hypertension [3] Normocapnia in severe TBI [4]
	Hypercapnia-induced increases in ICP	Avoid hypercapnia (PaCO_2_ > 45 mmHg) [3]
	Hypocapnia-induced reduction in CBF	Avoid severe (PaCO_2_ 26–31 mmHg), forced (PaCO_2_ < 26 mmHg) hypocapnia [3]
	High V_T_, continuous or short periods	Adjust V_T_ (6–8 mL/kg) according to predicted body weight [5]
	Low (0 cmH_2_O) or high PEEP (>8 cmH_2_O) levels	PEEP at 8, compared to 0 cmH_2_O, reduced the systemic inflammatory response in ABI patients [6]
	Occurrence of delirium	Check for key factors (age, cancer, sepsis, excessive use of sedative-hypnotic medication) [7,8] Hypoxic events should be avoided, but up to now, it is not directly linked to delirium [9] Antipsychotic agents remain the most common treatment [10]
Heart	ITP increases and may affect EDV and compliance of RV and LV	Evaluate RV and LV performance by non-invasive techniques, such as echocardiography. Check airway pressure and PEEP levels constantly [11,12]
	PEEP levels may increase PVR and RV afterload	Adjustment of PEEP with lung recruitment, no changes in PVR [13] Adjustment of PEEP with lung distension, PVR may increase [13]
Kidney	Elevated IAP may impair microvascular blood flow and venous drainage from kidneys	Check for other factors other than IMV (fluid balance, gastric distension) [14] Adjustment of PEEP to match IAP levels has shown positive results, as long no profound hemodynamic changes are observed [15]
	Increased risk of AKI	Protective V_T_ (6–8 mL/kg) was associated with more renal failure-free days [16] No association of low and high PEEP levels with renal failure-free days [17]

## Data Availability

Not applicable.

## Abbreviations

AKI	Acute kidney injury;
ARDS	Acute respiratory distress syndrome;
BiPAP	Bilevel positive airway pressure;
CBF	Cerebral blood flow;
COPD	Chronic obstructive pulmonary disease;
CPAP	Continuous positive airway pressure;
CPP	Cerebral perfusion pressure;
Eadyn	Dynamic central arterial elastance;
EBC	Exhaled breath condensate;
ETCO_2_	End-tidal CO_2_;
HFNO	High-flow nasal oxygen;
IAP	Intra-abdominal pressure;
ICP	Intracranial pressure;
IL	Interleukin;
IMV	Invasive mechanical ventilation;
ITP	Intrathoracic pressure;
LV	Left Ventricle;
NIV	Non-invasive ventilation;
PaCO_2_	Arterial partial pressure of carbon dioxide;
PEEP	Positive end-expiratory pressure;
PPV	Positive pressure ventilation;
PVR	Pulmonary vascular resistance;
RV	Right ventricle;
SVV	Systolic volume variation;
TBI	Traumatic brain injury;
TNF	Tumor necrosis factor;
V_T_	Tidal volume.
